# The Role of Uric Acid in Kidney Fibrosis: Experimental Evidences for the Causal Relationship

**DOI:** 10.1155/2014/638732

**Published:** 2014-05-05

**Authors:** Il Young Kim, Dong Won Lee, Soo Bong Lee, Ihm Soo Kwak

**Affiliations:** ^1^Division of Nephrology, Department of Internal Medicine, Pusan National University School of Medicine, Yangsan 626-770, Republic of Korea; ^2^Research Institute for Convergence of Biomedical Science and Technology, Pusan National University Yangsan Hospital, Yangsan 626-770, Republic of Korea; ^3^Medical Research Institute, Pusan National University Hospital, Busan 602-739, Republic of Korea

## Abstract

Hyperuricemia is a common finding in chronic kidney disease due to decreased uric acid clearance. The role of uric acid as a risk factor for chronic kidney disease has been largely debated, and recent studies suggested a role of uric acid in the causation and progression of kidney fibrosis, a final common pathway in chronic kidney disease. Uric acid and xanthine oxidase may contribute to kidney fibrosis mainly by inducing inflammation, endothelial dysfunction, oxidative stress, and activation of the renin-angiotensin system. Besides, hyperuricemia induces alterations in renal hemodynamics via afferent arteriolopathy and contributes to the onset and progression of kidney fibrosis. Xanthine oxidase inhibitors may prevent kidney damage via lowering uric acid and/or inhibiting xanthine oxidase. However, there is still no sufficient evidence from interventional clinical researches supporting the causal relationship between uric acid and kidney fibrosis. The effect and role of xanthine oxidase inhibitors in preventing kidney fibrosis and chronic kidney disease progression must be further explored by performing future large scale clinical trials.

## 1. Introduction

Regardless of the underlying etiology, most forms of chronic kidney disease (CKD) are characterized by progressive fibrosis as a final common pathway, which eventually affects all substructures of the kidney leading to a final consequence of end-stage renal disease. Although there has been a great deal of research, a comprehensive understanding of the pathogenetic mechanisms of kidney fibrosis remains uncertain and this hampers the development of effective therapeutic strategies [1].

Uric acid (UA) is the final breakdown product of purine degradation in humans, and elevated serum UA level, hyperuricemia, is causative in gout and urolithiasis due to the formation and deposition of monosodium urate crystals. Hyperuricemia is a common finding in CKD due to decreased UA clearance. Its role as a risk factor for CKD progression has been largely debated, and it was primarily considered as a marker or epiphenomenon of kidney damage [2, 3]. However, during the last 2 decades, accumulating evidences have suggested a role of UA in the causation or progression of cardiovascular diseases and CKD [3–9]. Therefore, UA lowering therapy with xanthine oxidase (XO) inhibitors, which are already being widely used in the treatment of gout, could be promising for preventing the progression of CKD even in patients without hyperuricemia; however, solid clinical evidence is still lacking. To promote large scale prospective clinical trials, it is essential to accumulate experimental evidences for the cause-effect relationship between UA and kidney fibrosis.

In this review, after providing a brief background concerning UA physiopathology, we will focus on the mechanistic role of UA in kidney fibrosis. We will also review the role of XO and the effect of XO inhibitors in preventing kidney fibrosis and their associated mechanisms.

## 2. Physiopathology of Uric Acid

**Figure 1:**
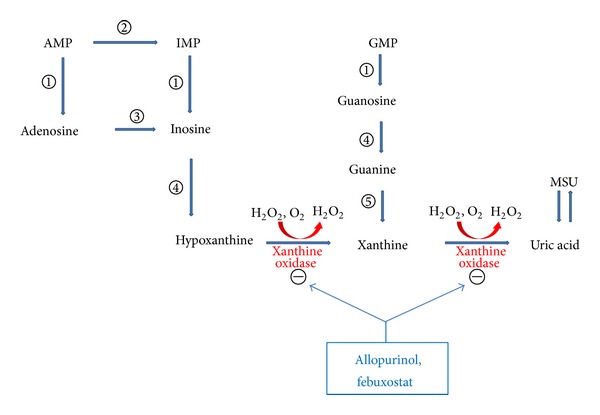
The pathway of purine nucleotides degradation in humans showing the competitive inhibition of uric acid formation by xanthine oxidase inhibitors and the site of action. AMP: adenosine monophosphate; GMP: guanosine monophosphate; IMP: inosine monophosphate; MSU: monosodium urate; *①*: 5′-nucleotidase; *②*: AMP deaminase; *③*: adenosine deaminase; *④*: purine nucleoside phosphorylase; *⑤*: guanine deaminase.

Cell turnover leads to the production of adenosine, inosine, and guanosine. They degrade to hypoxanthine and xanthine, which are the substrates for the widely distributed XO in the formation of UA. XO catalyzes the oxidation of purine substrates, xanthine and hypoxanthine, producing both UA and reactive oxygen species (ROS). Thus, XO is one of the major enzymatic sources of ROS. Allopurinol and febuxostat are inhibitors of XO, and they reduce uric acid and ROS formation (Figure 1) [10].

UA is the oxidation end-product of purine metabolism in humans and higher primates. Most other mammals, except for the Dalmatian dogs, can degrade UA further to water-soluble allantoin with the enzyme uricase, and as a result serum urate levels are about 10% of those in humans [9, 11]. However, in humans and higher primates, mutations in the uricase gene occurred during evolution and, making the enzyme nonfunctional, resulted in higher levels of serum UA than in other mammals [12].

Urates are the ionized form of UA, and, at a physiologic pH of 7.4, over 95% of UA dissociates into urates, with 98% existing as monosodium urate. The serum urate level depends on dietary purines, the breakdown of endogenous purines, and the renal and intestinal excretion of urate. Hyperuricemia is defined as the accumulation of serum UA beyond its solubility point in water (6.8 mg/dL), and it develops due to UA overproduction, undersecretion, or both [13]. The dominating factor contributing to hyperuricemia is underexcretion of urate [11]. Under normal conditions, 70% of the UA produced is eliminated in the urine and the remaining UA is removed via biliary secretion. In the kidney, urate is easily filtered through the glomerulus and subsequently reabsorbed by the proximal tubule cells of the kidney and after further absorption, about 10% of urate is finally excreted [14]. An anion exchanger and a voltage-dependent pathway seem to be the mechanisms involved in urate transport [15].

Allopurinol, a purine inhibitor of XO, has been conventionally used for urate-lowering therapy to inhibit UA synthesis. A novel urate-lowering drug, febuxostat, is a potent nonpurine selective inhibitor of XO, and it inhibits both the reduced and oxidized forms of the enzyme in contrast to allopurinol that inhibits the reduced form of the enzyme only [16, 17]. Febuxostat is metabolized mainly by glucuronidation and oxidation in the liver, has its dual (urinary and fecal) pathways in excretion (urinary and fecal excretion rates: 49.1% and 44.9%, resp.), and is effective and well tolerated in patients with mild to moderate renal and hepatic impairment [18, 19]. Animal studies have demonstrated that febuxostat has a greater UA-lowering effect than allopurinol [20, 21]. Febuxostat's chemical structure does not resemble a pyrimidine or purine and is unlike that of allopurinol [22]. It does not inhibit other enzymes involved in purine or pyrimidine metabolism [23].

Studying the role of UA in kidney fibrosis, a process which eventually leads to CKD, is very difficult since uric acid is excreted primarily by the kidney, and hence a decrease in the glomerular filtration rate (GFR) is inevitably accompanied by a rise in the serum UA level. As such, studies in experimental animals in which serum UA can be modulated are critical to understand whether there is a role for UA in the causation or progression of CKD [7].

## 3. Experimental Studies Supporting the Roles of Uric Acid and Xanthine Oxidase in Kidney Fibrosis 

In the past, hyperuricemia was thought to cause CKD, the so-called urate nephropathy, by the deposition of urate crystals in the renal interstitium. This results in a chronic inflammatory response and in progressive tubulointerstitial injury in a similar manner as seen with tophi in gouty arthritis [24]. However, the pathologic role of hyperuricemia in kidney disease by a crystal-independent mechanism is somewhat less clear.

### 3.1. Hyperuricemic Rat Models and Types of Kidney Injury

Generating hyperuricemia in laboratory animals proved to be difficult due to the fact that most mammals have the uricase enzyme. Rodent models in which the uricase gene was knocked out showed renal failure due to extensive tubular crystal deposition and finally death [25]. An alternative model with milder degree of hyperuricemia without crystal deposition, which is more applicable to human disease, was developed using uricase inhibitor, oxonic acid (OA) [26–29]. It is also possible to lower serum UA levels using the XO inhibitors such as allopurinol and febuxostat.

After the year 2001, when the hyperuricemic rat model was developed by using OA [26] and until recently, there have been accumulating experimental evidences that hyperuricemia induced renal injury, which may be prevented by lowering serum UA levels with XO inhibitors. The first study using OA-induced hyperuricemic rat model demonstrated that hyperuricemia induced systemic hypertension as well as ischemic type of kidney injury with collagen deposition, macrophage infiltration, and increase in tubular expression of osteopontin documented via immunohistochemical stains. The kidneys were devoid of urate crystals and were normal by light microscopy. Blood pressure was lowered by reducing serum UA levels with allopurinol. Hyperuricemic rats treated with OA also showed an increase in juxtaglomerular renin and a decrease in macula densa neuronal nitric oxide (NO) synthase. Both the kidney injury and hypertension were attenuated by treatment with renin-angiotensin system (RAS) blocker (enalapril) or a substrate for endothelial NO synthase (L-arginine). This study suggested that UA induced hypertension and renal injury via a crystal-independent mechanism with the activation of RAS and inhibition of NO synthase [26]. Another study demonstrated that hyperuricemia induced arteriolopathy of the afferent arteriole by a blood pressure-independent mechanism. In this study, hyperuricemic rats fed OA showed hypertension and afferent arteriolar thickening. Allopurinol prevented hyperuricemia, hypertension, and arteriolopathy. Controlling blood pressure with hydrochlorothiazide did not prevent hyperuricemia and arteriolopathy, suggesting that hyperuricemia-induced arteriolopathy was not mediated by blood pressure. This study also showed that arteriolopathy was mediated by the direct effect of UA on proliferation of vascular smooth muscle cells with activation of RAS [27].

In another rat model with dietary intake of adenine, which may be the source of the UA as a purine base, adenine-fed rats showed hyperuricemia [30, 31]. Adenine-fed rats also showed increased kidney inflammation (TNF-*α*), fibrotic (TGF-*β*), and oxidative (HO-1) markers, along with pathologically confirmed kidney fibrosis. Lowering of UA levels with allopurinol reversed the kidney damage, suggesting that UA played a major role in the pathogenesis of kidney fibrosis [30]. Another animal model of tubulointerstitial nephritis (TIN) induced by excessive adenine intake exhibited significant renal dysfunction and enhanced cellular infiltration accompanied by collagen deposition. It also showed higher gene and protein expression of proinflammatory cytokines. Treatment with allopurinol led to reduced levels of uric acid, oxidative stress, and collagen deposition and a downregulation of the nuclear factor-kB (NF-kB) signaling pathway [31].

Based on the growing evidence that lowering UA levels with allopurinol prevented renal injury induced by hyperuricemia, the role of febuxostat in preventing kidney injury was investigated. In OA-induced hyperuricemic rats, febuxostat lowered UA levels and ameliorated systemic and glomerular hypertension as well as preglomerular arteriolopathy. In normal rats without hyperuricemia, febuxostat tended to lower UA levels and had no effect on blood pressure, glomerular pressure, and afferent arteriole morphology [32].

### 3.2. Systemic and Glomerular Hypertension in Hyperuricemia

There are some experimental studies that demonstrated the relationship between UA and renal hemodynamics. In one study, hyperuricemic rats fed OA not only developed systemic hypertension but also glomerular hypertension. Hyperuricemic rats showed increased glomerular capillary pressure with afferent arteriole thickening. Allopurinol prevented hyperuricemia, systemic and glomerular hypertension, and arteriolopathy. Glomerular capillary pressure and arteriolar thickening correlated with serum UA and systolic blood pressure. This study suggested that glomerular hypertension might be mediated by insufficient vasoconstriction of the afferent arteriole to systemic hypertension, allowing the transmission of systemic pressure to the glomerular capillary tuft [33]. In another study, hyperuricemia induced by OA resulted in renal cortical vasoconstriction and glomerular hypertension due to afferent arteriole thickening in normal and remnant kidney rats. Allopurinol prevented structural and functional alterations in both normal and remnant kidney rats. This study suggested that hyperuricemia-induced glomerular alterations caused renal ischemia, which in turn induced tubulointerstitial inflammation and fibrosis [28].

### 3.3. Renal Progression in Animal Models of Chronic Kidney Disease

Hyperuricemia also contributed to renal progression in animal models of CKD. In 5/6 nephrectomy remnant kidney model, remnant kidney rats fed OA showed more severe renal failure, proteinuria, and histologic findings (thickening of the preglomerular arteries, glomerulosclerosis, and interstitial fibrosis) compared to remnant kidney rats without hyperuricemia. Allopurinol reduced serum UA levels and prevented the functional and histologic changes in remnant kidney rats fed OA. This study also demonstrated that hyperuricemia accelerated renal progression by increasing renin expression in the renal cortex and cyclooxygenase-2 (COX-2) expression in the afferent arteriole. In particular, this study showed that increased COX-2 expression induced by hyperuricemia was associated with proliferation of vascular smooth muscle cells in preglomerular arteries [29]. In another 5/6 nephrectomy rat model, remnant kidney rats treated with OA developed hyperuricemia, renal vasoconstriction, and glomerular hypertension in association with further aggravation of afferent arteriolopathy compared to remnant kidney rats that were not treated with OA. Febuxostat prevented hyperuricemia and ameliorated renal injury in remnant kidney rats treated with OA. Interestingly, febuxostat had a comparable beneficial effect in both remnant kidney rats with hyperuricemia (treated with OA) and remnant kidney rats without hyperuricemia (not treated with OA) [34].

### 3.4. Exacerbation of Renal Injury in Animal Models of Cyclosporine and Diabetic Nephropathy

Hyperuricemia has also been known to exacerbate renal injury in some animal disease models including cyclosporine (CsA) and diabetic nephropathy. Hyperuricemia frequently complicated CsA therapy. In one study using a model of CsA nephropathy, the rats developed hyperuricemia with arteriolar hyalinosis, tubular injury, and interstitial fibrosis. CsA nephropathy rats fed OA showed higher UA levels with more severe histologic findings compared to CsA nephropathy rats that were not treated with OA. This study also demonstrated that the mechanism did not involve intrarenal urate crystal deposition and appeared to involve activation of RAS and inhibition of intrarenal NO production [35]. In another study, CsA-treated rats developed hyperuricemia with arteriolar hyalinosis, tubular atrophy, interstitial fibrosis, increased cell proliferation, and decreased vascular endothelial growth factor (VEGF). Treatment with allopurinol or a uricosuric, benzbromarone, reduced the severity of the kidney injury. Both drugs provided comparable protection and the similar protection observed with both drugs suggests that the effect is associated more with lowering UA levels than the antioxidant effect of allopurinol [36]. Hyperuricemia has recently been recognized to be a risk factor for nephropathy in the diabetic subject. Diabetic (db/db) mice developed hyperuricemia, albuminuria, mesangial matrix expansion, and mild tubulointerstitial disease. Allopurinol treatment not only reduced UA levels but also reduced albuminuria and ameliorated tubulointerstitial injury. The mechanism for protection was shown to be due to a reduction in inflammatory cells mediated by a reduction in ICAM-1 expression by tubular epithelial cells [37]. In another diabetic nephropathy model using KK-A(y)Ta mice, lowering UA levels with allopurinol attenuated transforming growth factor- (TGF-) *β*1-induced profibrogenic progression in the mice, suggesting that lowering serum UA may be an effective therapeutic intervention to prevent the progression of diabetic nephropathy [38].

### 3.5. Oxidative Stress and Endothelial Dysfunction in Hyperuricemia

While UA has been reported to be a potent antioxidant in the extracellular fluid [39], it has prooxidative effect once inside the cell [40, 41]. According to a hypothesis [39], the silencing of the uricase gene with an increase in the blood level of UA provided an evolutionary advantage for ancestors of* Homo sapiens*. This hypothesis was based on* in vitro* experiments which showed that UA is a powerful scavenger of singlet oxygen, peroxyl radicals, and hydroxyl radicals. UA circulating at an elevated level was proposed to be one of the major antioxidants of the plasma that protects cells from oxidative damage, thereby contributing to an increase in life span of human species and decreasing the risk of cancer [42]. On the other hand, a vast literature on the epidemiology of cardiovascular disease, hypertension, and metabolic syndrome overwhelmingly shows that, at least among modern* Homo sapiens*, a high level of UA is strongly associated with and in many cases predicts development of hypertension, visceral obesity, insulin resistance, dyslipidemia, diabetes, kidney disease, and cardiovascular and cerebrovascular events [42]. Antioxidant effect of UA varies according to the presence of specific components, in different physiochemical circumstances and in various compartments of the human body. UA is an antioxidant only in the hydrophilic environment and even in the plasma UA can prevent lipid peroxidation only as long as ascorbic acid is present [43]. Major sites where the antioxidant effects have been proposed are the central nervous system [44, 45], liver [46], and heart [47].

In OA-induced hyperuricemic rat model, hyperuricemia caused intrarenal oxidative stress, increased expression of NOX-4 subunit of renal NADPH oxidase and angiotensin II, and decreased NO bioavailability. Hyperuricemic rats also showed systemic hypertension, renal vasoconstriction, and arteriolopathy. Tempol (superoxide scavenger) attenuated the adverse effect induced by hyperuricemia despite equivalent hyperuricemia, suggesting that UA might cause oxidative stress [48]. In another study, hyperuricemic rats treated with OA showed decreased urinary NO metabolites (NO_2_^−^/NO_3_^−^), systemic hypertension, renal vasoconstriction, and preglomerular arteriolopathy. Chronic administration of L-arginine, a substrate for endothelial NO synthase, increased the urinary excretion of NO_2_^−^/NO_3_^−^ and preserved arteriolar structures probably mediated by the antiproliferative effect of NO on vascular smooth muscle cells, suggesting the role of endothelial dysfunction as a mediator of renal injury induced by hyperuricemia [49]. A recent study reported that UA-induced endothelial dysfunction was associated with mitochondrial alterations and decreased intracellular ATP concentrations [50]. An experimental model of streptozotocin-induced diabetic rats showed that febuxostat improved endothelial dysfunction via attenuating oxidative stress by XO inhibition [51].

### 3.6. Hyperuricemia and Inflammatory Responses

UA induces inflammatory responses. Hyperuricemia-induced inflammatory response mediates kidney injury via alteration of vascular and tubular cells in kidney. UA has the ability to induce monocyte chemoattractant protein- (MCP-) 1 in vascular smooth muscle cells, suggesting that it may have a role in the vascular changes associated with hypertension and vascular disease [52]. UA also contributes to kidney damage through vascular cell proliferation induced by activation of COX-2 [29] and increased expression of C-reactive protein (CRP) [53]. UA has been known to inhibit renal proximal tubule cell proliferation via activation of NF-*κ*B and cytoplasmic phospholipase A_2_ [54]. Hyperuricemia also increases extracellular matrix (ECM) synthesis through upregulation of lysyl oxidase (LOX) expression in renal tubular epithelial cells [55]. UA contributes to tubulointerstitial inflammation by inducing expression of intracellular adhesion molecule- (ICAM-) 1 in renal tubular epithelial cells [37].

XO has been reported to be upregulated by various inflammatory stimuli such as lipopolysaccharide (LPS), hypoxia, and cytokines [56–60]. Augmented XO eventually causes excess ROS formation, leading to tissue damage. Pharmacological inhibitors of XO, such as allopurinol and febuxostat, have been reported to have an anti-inflammatory effect in various diseases such as atherosclerosis, congestive heart failure, acute lung injury, renal interstitial fibrosis, and ischemia-reperfusion injury [61–66]. A recent study suggested a molecular mechanism underlying the involvement of XO in inflammatory pathways, and it also suggested that XO mediates LPS-induced phosphorylation of JNK through ROS production and MKP-1 inactivation, leading to MCP-1 production in macrophages. Febuxostat significantly suppressed LPS-induced MCP-1 production in human macrophages and* in vivo* in mice [56].

### 3.7. Hyperuricemia and Epithelial-Mesenchymal Transition

In the last decade, epithelial-mesenchymal transition (EMT), a process by which fully differentiated epithelial cells lose their epithelial characteristics and undergo phenotypic conversion to mesenchymal cells, has emerged as an important pathway leading to generation of matrix-producing fibroblasts and myofibroblasts in kidney fibrosis. In addition to kidney fibrosis, EMT has been known to play a pivotal role in embryonic development, wound healing, tissue regeneration, and cancer progression [67, 68].

A recent study showed that UA exerted a direct effect on renal tubular cells by inducing EMT [69]. OA-induced hyperuricemic rats showed evidence of EMT before the development of significant tubulointerstitial fibrosis at 4 weeks, as indicated by decreased E-cadherin expression and an increased *α*-smooth muscle actin (*α*-SMA). Allopurinol significantly inhibited UA-induced changes in E-cadherin and *α*-SMA with an amelioration of kidney fibrosis at 6 weeks. In cultured rat renal tubular epithelial cells (NRK cells), UA induced EMT, which was blocked by the organic acid transport inhibitor, probenecid. UA increased expression of transcriptional factors associated with decreased synthesis of E-cadherin. UA also increased the degradation of E-cadherin via ubiquitination, which is of importance since downregulation of E-cadherin is considered to be a triggering mechanism for EMT. This study suggested that UA-induced EMT of renal tubular cells might be a novel mechanism explaining the association of hyperuricemia and renal progression after taking into account that EMT is an early phenomenon in kidney fibrosis [69–71]. Further research is required to assess the role of UA in other mesenchymal cell generation pathways.

## 4. Perspective

The underlying mechanisms by which UA could cause kidney fibrosis have been reported in animal models since 2001, and there has been a growing interest in this topic and numerous retrospective and prospective observational studies have been performed to assess the relationship between UA and CKD. The majority of data support the role of UA as a cause or exacerbating factor for kidney fibrosis and progressive CKD. Although, over the last several years, some clinical intervention trials including randomized controlled trials (RCTs) have further supported this mechanistic role of UA [72–78], clinical evidence demonstrating the beneficial effect of UA lowering therapy with XO inhibitors on renoprotection and prevention of CKD progression is not certain yet and cannot be easily generalized. Most of the clinical studies are limited by their retrospective study design, small sample size, short follow-up duration, or lack of randomization, and no adequately powered RCT has yet demonstrated the beneficial effect of UA-lowering therapy on renal and cardiovascular outcomes in CKD [5]. Currently, there is no published clinical trial in which febuxostat was used as a XO inhibitor for evaluating the efficacy of UA reduction on progression of CKD.

With regard to the animal models in this research field, the uricase inhibitor OA-induced hyperuricemic rat model [26–29] was mainly used during the initial several years and all of the studies used allopurinol as a XO inhibitor. Thereafter, the research was performed using animal models of various kidney disease conditions, such as CsA nephropathy [36], diabetic nephropathy [38, 51], obstructive nephropathy [61], ischemia-reperfusion injury [62], and 5/6 nephrectomy rat model [34]. Further research using diverse animal models of CKD [79, 80] is essential not only for gaining a better understanding of molecular mechanisms of kidney fibrosis but also for designing more appropriate clinical studies.

Febuxostat, a chemically engineered nonpurine selective inhibitor of XO, received approval in February 2009 from the Food and Drug Administration for the chronic management of hyperuricemia in patients with gout [81]. Although its clinical use is increasing, febuxostat is still generally considered as a second-line option for patients with gout who are unable to take allopurinol due to hypersensitivity, intolerance, renal insufficiency, or lack of efficacy in achieving a target serum UA level of <6.0 mg/dL. However, recently in basic research, febuxostat has largely replaced allopurinol as a XO inhibitor, and several animal studies have shown various beneficial effects of febuxostat in reducing inflammation, oxidative stress, and kidney fibrosis [34, 61, 62]. Febuxostat prevented renal injury in 5/6 nephrectomy rats with and without coexisting hyperuricemia [34]. But still there is little clinical experience and there are no clinical trials with febuxostat assessing the efficacy of lowering UA on CKD progression, and it is also more expensive than allopurinol.

At present, drug therapy for asymptomatic hyperuricemia is not actively recommended, and this negative approach is mainly due to the absence of evidence from adequately powered RCTs on the causality between hyperuricemia and the onset or progression of CKD. Considering the large sample size required for an adequately powered trial, an international collaboration is necessary.

Current research interests focus on developing new effective antifibrotic drugs to slow progression or even reverse chronic kidney injury, and several compounds which target various components of the fibrotic pathway, from signaling molecules that include TGF-*β*, phosphatidylinositide-3-kinase, and chemokines to microRNAs, are undergoing clinical trials [82, 83]. Although, at present, UA may be one of the ignored risk factors for CKD and the clinical use of UA-lowering drugs which include allopurinol and febuxostat is largely confined to gout management, discovering the potential value of XO inhibitors for preventing kidney fibrosis and CKD progression will provide additional valuable tools for managing CKD.

## 5. Conclusion

**Figure 2:**
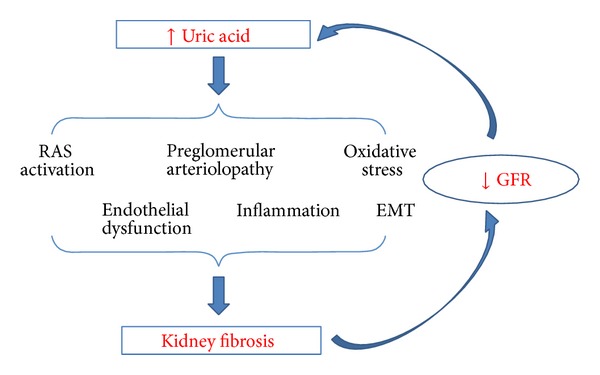
Mechanisms by which uric acid may cause kidney fibrosis based on experimental animal studies. EMT: epithelial-mesenchymal transition; RAS: renin-angiotensin system.

After taking into account the results of all the important experimental studies mentioned above, UA and XO may contribute to kidney fibrosis mainly by inducing inflammation, endothelial dysfunction, oxidative stress, and activation of RAS (Figure 2). Besides, hyperuricemia induces alterations of renal hemodynamics via afferent arteriolopathy and contributes to the onset and progression of kidney fibrosis. Many experimental studies have shown that XO inhibitors may prevent kidney damage by lowering UA and inhibiting XO. However, there is no sufficient evidence from interventional clinical researches supporting the causal relationship between UA and kidney fibrosis, a final common pathway of CKD progression. Thus, the time is not quite appropriate for recommending the widespread clinical use of XO inhibitors for preventing CKD progression in patients with hyperuricemia and more so in patients without hyperuricemia. The effect and role of XO inhibitors in preventing kidney fibrosis and CKD progression must be further explored by performing future large scale clinical trials.
